# Simultaneous lasing of Ni *K*α and Cu *K*α lasers from an alloy foil irradiated with an intense X-ray free-electron laser pulse

**DOI:** 10.1107/S1600577525010914

**Published:** 2026-01-20

**Authors:** Yuichi Inubushi, Gota Yamaguchi, Jumpei Yamada, Yuya Kubota, Ichiro Inoue, Taito Osaka, Makina Yabashi

**Affiliations:** a Japan Synchrotron Radiation Research Institute 1-1-1 Kouto, Sayo-cho Sayo-gun Hyogo679-5198 Japan; b RIKEN SPring-8 Center, 1-1-1 Kouto, Sayo-cho, Sayo-gun, Hyogo679-5148, Japan; c Graduate School of Engineering The University of Osaka 2-1 Yamada-oka Suita Osaka565-0871 Japan; RIKEN SPring-8 Center, Japan

**Keywords:** X-ray free-electron laser, *K*α laser, single-shot X-ray spectroscopy

## Abstract

We report the simultaneous lasing of two distinct *K*α emissions at photon energies of 7.48 keV (Ni *K*α_1_) and 8.05 keV (Cu *K*α_1_). This was achieved by a population inversion induced through intense X-ray free-electron laser (XFEL) irradiation of a Cu–Ni alloy foil. This demonstration of multi-color X-ray lasing using a single XFEL source is expected to contribute significantly to the future development of X-ray lasers and their applications.

## Abstract 2

We successfully achieved simultaneous lasing of 7.48 keV Ni *K*α_1_ and 8.05 keV Cu *K*α_1_ emissions using an intense X-ray free-electron laser (XFEL) pulse. This achievement – the realization of simultaneous two-color X-ray lasing using a single-color XFEL pulse – is expected to advance the development of X-ray lasers and their applications.

## 1. Introduction

Brilliant femtosecond X-ray free-electron laser (XFEL) pulses (Emma *et al.*, 2010 ▸; Ishikawa *et al.*, 2012 ▸) have played an important role in opening up new fields of X-ray science (Chapman *et al.*, 2011 ▸; Suga *et al.*, 2015 ▸; Kim *et al.*, 2020 ▸). Lasing via amplified spontaneous emission (ASE) of *K*α emission has been successfully achieved by generating population inversion through high-intensity XFEL irradiation of various materials (Rohringer *et al.*, 2012 ▸; Yoneda *et al.*, 2015 ▸; Kroll *et al.*, 2018 ▸; Kroll *et al.*, 2020 ▸; Zhang *et al.*, 2022 ▸; Doyle *et al.*, 2023 ▸; Linker *et al.*, 2025 ▸). If ASE lasing at different wavelengths can be simultaneously induced using a single XFEL pulse, multi-color X-ray lasers could be realized. Such a capability is expected to greatly advance the development of X-ray laser systems and enable novel applications, including laser oscillators (Halavanau *et al.*, 2020 ▸) on multi-color X-rays and nonlinear X-ray spectroscopic techniques (Tanaka & Mukamel, 2002 ▸; Sun *et al.*, 2010 ▸), which require synchronized multi-color X-ray sources.

When a material is irradiated with X-rays, various characteristic emissions such as *K*α_1_, *K*α_2_ and *K*β lines are simultaneously generated. In spontaneous emission, the intensity of *K*α_1_ is typically about twice that of *K*α_2_. However, in the case of stimulated emission, such as in ASE X-ray lasers, the transition from the 2*p*_3/2_ to the 1*s* state (which has a significantly larger stimulated emission cross section than the 2*p*_1/2_ to 1*s* transition responsible for *K*α_2_) dominates. As a result, only *K*α_1_ typically undergoes lasing. When excited by extremely intense X-rays, *K*α_2_ lasing may occur very weakly like a satellite of *K*α_1_ (Yoneda *et al.*, 2015 ▸). Similarly, *K*β ASE lasing is nearly impossible due to strong competition with *K*α transitions. These constraints make the generation of multi-color ASE X-ray lasers from a single-element sample extremely challenging. To overcome this, we propose using a sample composed of different atomic species to achieve simultaneous multi-color ASE lasing. Since ASE lasing occurs independently in each atomic species, simultaneous oscillation of distinct *K*α_1_ lines becomes possible. In this study, we report the simultaneous generation of 7.48 keV Ni *K*α_1_ and 8.05 keV Cu *K*α_1_ ASE lasers using a foil of Ni–Cu alloy.

## 2. Experiment and discussion

**Figure 1:**
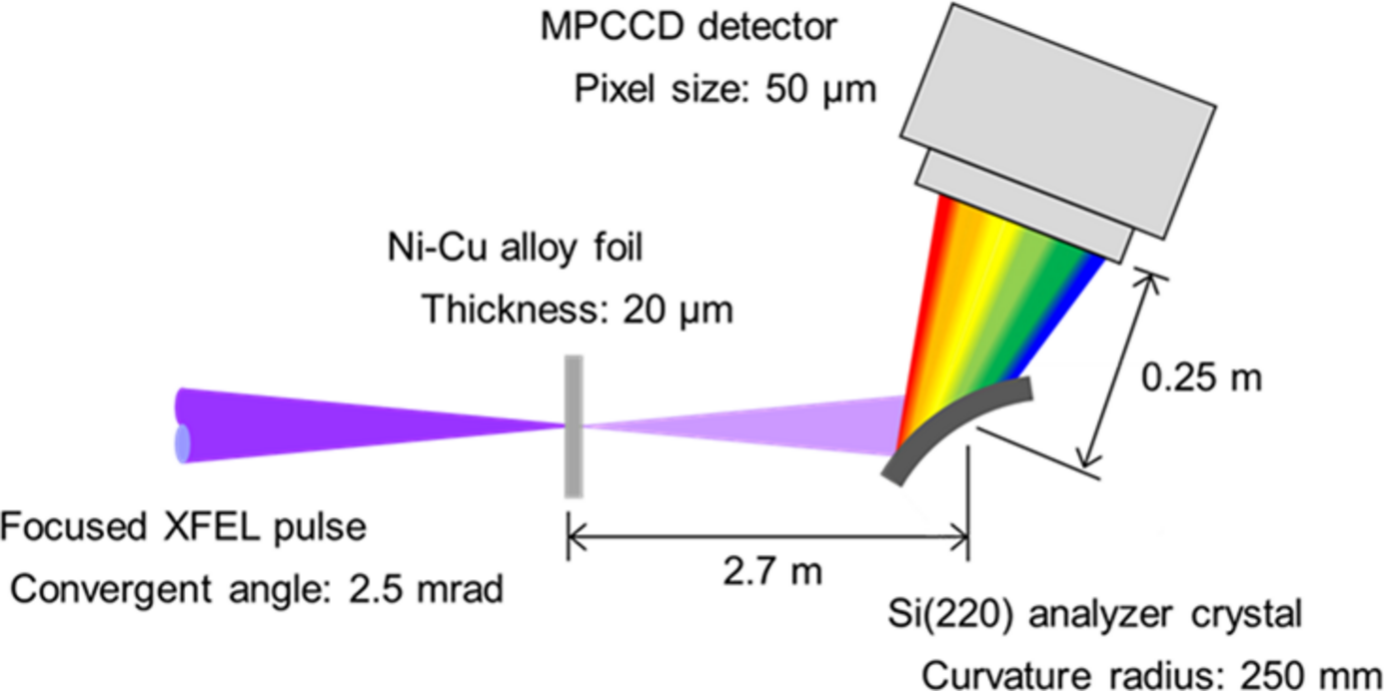
Schematic of the experimental setup. Focused 10 keV XFEL pulses irradiated a Ni–Cu alloy (constantan) foil. The resulting *K*α_1_ lasers were simultaneously measured in a single-shot manner using a dispersive spectrometer consisting of a convex bent Si(220) analyzer crystal and an MPCCD detector. The central photon energy for the observation was set to 7.8 keV, corresponding to a Bragg angle of 24.5°. An energy observation range of 1.2 keV was achieved, with an energy resolution of 1.5 eV per pixel.

The experiment was conducted at SACLA BL2 EH3. A schematic of the experimental setup is shown in Fig. 1 ▸. XFEL pulses with a photon energy of 10 keV were focused using Kirkpatrick–Baez (KB) optics (Inubushi *et al.*, 2025 ▸). The beam sizes were measured to be 150 nm (horizontal) and 220 nm (vertical) in full width at half-maximum (FWHM). Typically, a pulse energy of 110 µJ was achieved at the focal point. The pulse duration was 7 fs (Inubushi *et al.*, 2012 ▸; Inubushi *et al.*, 2017 ▸), resulting in an intensity of 2.5 × 10^19^ W cm^−2^. The XFEL pulses were focused onto a 20 µm-thick constantan foil, an alloy composed of 45% Ni and 55% Cu. Given that the *K*-absorption edges of Ni and Cu are 8.33 keV and 8.98 keV, respectively, the 10 keV XFEL pulses were capable of ionizing the *K*-shell electrons of both elements. The constantan foil, placed in air, was moved after each shot to ensure irradiation on a fresh surface. The XFEL intensity was varied by adjusting the position of the foil along the beam path. In this context, the position where the maximum XFEL intensity was achieved is referred to as the ‘optimal position’.

To verify the simultaneous lasing of two *K*α_1_ lines, a single-shot measurement with an energy observation range exceeding 600 eV is necessary, as the photon energies of Ni *K*α_1_ and Cu *K*α_1_ emissions are 7.48 keV and 8.05 keV, respectively. Here, we propose the combined use of a divergent X-ray beam and a bent crystal (Zhu *et al.*, 2012 ▸) to achieve a significantly broader energy observation range. In our experiment, a convex bent Si(220) crystal with a curvature radius of 250 mm was employed. A ray-tracing simulation was conducted to design and optimize the spectrometer configuration. Fig. 1 ▸ shows the experimental setup, including the parameters used in the ray-tracing simulation. As a result, an energy observation range of 1.2 keV was achieved. The MPCCD detector (Kameshima *et al.*, 2014 ▸) provided an energy resolution of 1.5 eV per pixel.

**Figure 2:**
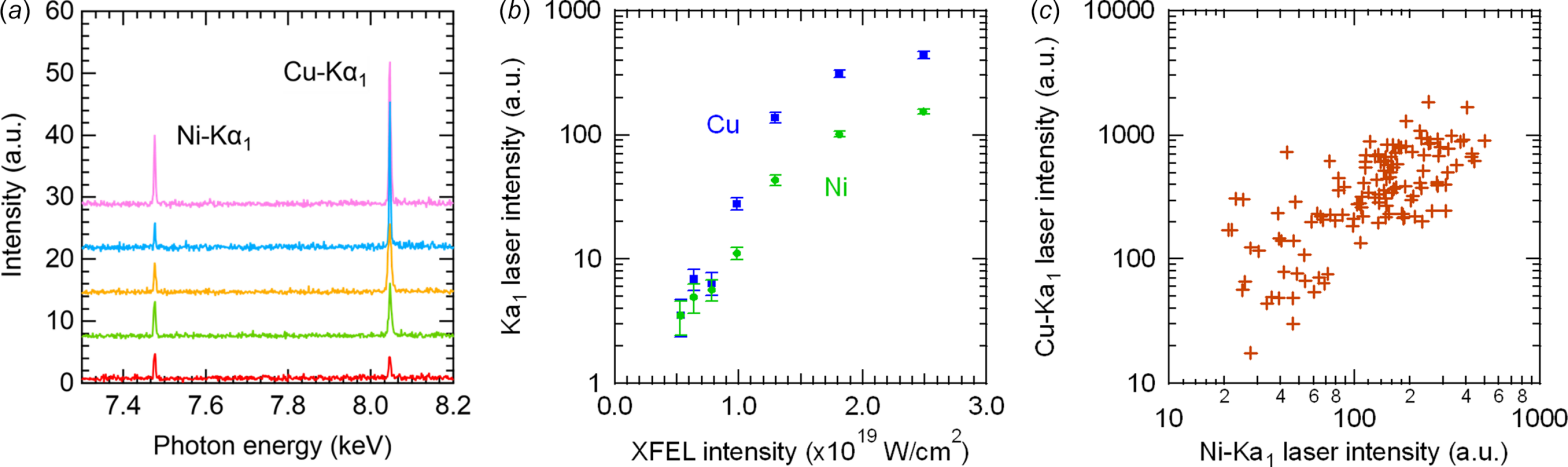
(*a*) Single-shot spectra observed at the optimal position. (*b*) Averaged intensities of the Ni *K*α_1_ and Cu *K*α_1_ lasers as functions of XFEL intensity. Error bars represent standard errors. (*c*) Correlation between the intensities of the Ni *K*α_1_ and Cu *K*α_1_ lasers at the optimal position.

Fig. 2 ▸(*a*) shows the single-shot spectra measured at the ‘optimal position’. Distinct peaks are observed at 7.48 keV, corresponding to the Ni *K*α_1_ line, and at 8.05 keV for the Cu *K*α_1_ line, with variations in intensity. As illustrated in Fig. 2 ▸(*b*), both peaks exhibit nonlinear increases in signal intensity as a function of XFEL intensity, indicating successful simultaneous lasing of the Ni *K*α_1_ and Cu *K*α_1_ lines. At higher XFEL intensities, the Cu *K*α_1_ laser becomes more intense than the Ni *K*α_1_ laser. This is likely due to the higher Cu content in the alloy, the greater absorption coefficient of Cu for 10 keV X-rays and the higher transmittance of the Cu *K*α_1_ emission through the sample. Furthermore, as shown in Fig. 2 ▸(*c*), a statistically significant positive correlation was observed between the Ni *K*α_1_ and Cu *K*α_1_ laser intensities (correlation coefficient *r* = 0.63, *p* value *p* < 0.001), indicating that the two lasing processes are not mutually competitive. This implies that increasing the XFEL beam intensity could lead to the simultaneous enhancement of both lasers, until saturation.

## 3. Summary and future perspectives

We successfully demonstrated the simultaneous generation of two *K*α_1_ lasers by irradiating an alloy foil with an intense XFEL pulse. This achievement suggests the potential for producing multi-color X-ray laser pulses by incorporating multiple atomic species – not limited to alloys, but also including, for example, layered sample and mixed-atom droplets. Such multi-color X-ray lasers are expected to pave the way for future advancements in X-ray laser technology and enable novel applications across various scientific fields.
